# Optimal upfront surgery for gastric adenocarcinoma. Real life situation in Brazil. Results comparable to neoadjuvant treatment

**DOI:** 10.1590/0102-67202025000055e1924

**Published:** 2026-02-13

**Authors:** Augusto Canton GONÇALVES, Rodrigo Silveira RACY, Anna Clara Hebling MITIDIERI, Beny Goulart Dias de CASTRO, Caio de Carvalho ZANON, Wilson Rodrigues FREITAS, Osvaldo Antonio Prado CASTRO, Paulo KASSAB

**Affiliations:** 1 Faculdade de Ciências Médicas da Santa Casa de São Paulo, Department of Surgery – São Paulo (SP), Brazil.

**Keywords:** Stomach neoplasms, Gastrectomy, Neoadjuvant therapy, Gastric outlet obstruction, Neoplasias gástricas, Gastrectomia, Terapia neoadjuvante, Obstrução da saída gástrica

## Abstract

**Background:**

Complete neoadjuvant treatment for gastric cancer is not always tolerated due to nutritional and clinical reasons, such as gastric outlet obstruction. In this context, upfront surgery becomes an alternative.

**Aims:**

The aim of the study was to compare upfront resection with neoadjuvant systemic therapy followed by surgery and identify factors influencing their outcomes.

**Methods:**

Retrospective study of 410 patients with locally advanced gastric adenocarcinoma followed between 2012 and 2020, comparing upfront surgery and perioperative treatment. Patients with early tumor (cT1N0), metastasis, and stump cancer were excluded. The comparison was stratified by stage without the influence of systemic treatment (primary stage). Resections with D2 dissection, no residual tumor (no R2), and no complications were considered optimal surgery.

**Results:**

Upfront resection was performed in 216 patients (85% of upfront surgeries). Gastrectomy after neoadjuvant treatment was performed in 47 cases (76% of indications), and another four were resected among 39 previous unsuccessful surgeries (10%). In total, there were 51 resections after chemotherapy. Independent factors associated with overall survival at 60 months were: preoperative chemotherapy (57.3% vs. 40.7%, p=0.029); complication rate; D2 lymphadenectomy; and primary stage. Initial cases showed a better outcome in the neoadjuvant group without statistical significance (p=0.447), but it was present in more advanced tumors (p=0.027). Optimal surgery was achieved in 68.6% of the neoadjuvant group and 51.9% of the upfront group (p=0.030) and resulted in similar overall survival (56.6% vs. 52.4%, p=0.904).

**Conclusions:**

Optimal upfront surgery followed by adjuvant therapy, particularly with D2 dissection, is effective and was not statistically inferior to neoadjuvant treatment.

## ARTICLE HIGHLIGHTS

Preoperative systemic treatment is indicated for locally advanced gastric cancer in Western countries.

However, especially in obstructive tumors, it is not tolerated.

Optimal upfront surgery — radical and without complications — has demonstrated similar results to perioperative treatment.

## Summary

**CENTRAL MESSAGE:**

The distal stomach remains an important site of gastric adenocarcinoma. Due to the high rate of late diagnoses, patients’ nutritional status is commonly affected, sometimes with gastric outlet obstruction, which often requires intervention before systemic treatment. Patient morbidity, costs, side effects, and variable response rates also hinder the decision for neoadjuvant treatment. In this context, upfront surgery becomes an alternative.

**PERSPECTIVES:**

The higher incidence of distal tumors and subtotal gastrectomies in the UPF group, within a context of more advanced stages, might suggest a higher proportion of obstructive tumors and patients with poorer baseline status. However, even in this context, the outcomes of optimal upfront surgery were similar to those of neoadjuvant treatment. Optimal upfront surgery followed by adjuvant therapy, particularly with D2 dissection, is effective and was not statistically inferior to neoadjuvant treatment.

## VISUAL ABSTRACT

**Figure:**
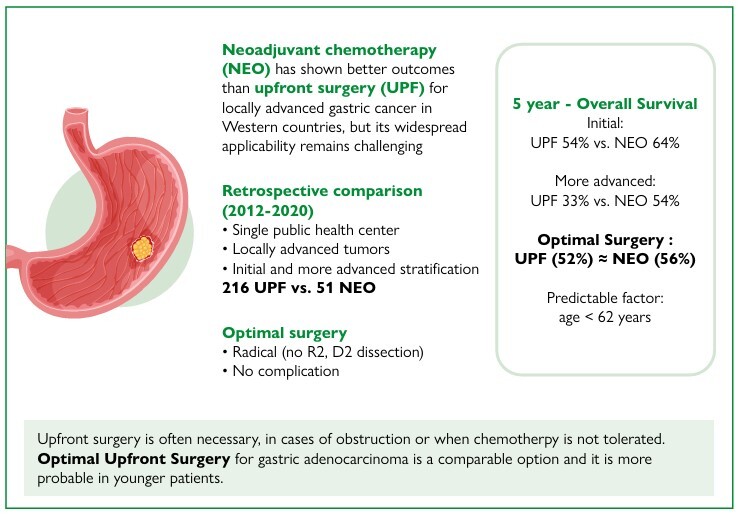

## RESUMO

**Racional:**

O tratamento neoadjuvante completo para o câncer gástrico nem sempre é tolerado por razões nutricionais e clínicas, como obstrução antropilórica. Nesse contexto, a cirurgia de princípio torna-se uma alternativa.

**Objetivos:**

Comparar ressecção de princípio com terapia sistêmica neoadjuvante, e identificar fatores que influenciam os resultados.

**Métodos:**

Estudo retrospectivo entre 2012 e 2020, de 410 pacientes com tumores localmente avançados. Lesões precoces (cT1N0), metastáticas e câncer de coto gástrico foram excluídos. O estadiamento foi estratificado sem influência do tratamento sistêmico (estadiamento primário). Ressecções com dissecção D2, sem tumor residual (não R2) e sem complicações foram consideradas cirurgias ótimas.

**Resultados:**

A ressecção de princípio foi realizada em 216 pacientes (85% das cirurgias de princípio). A gastrectomia após o tratamento neoadjuvante ocorreu em 47 casos (76% das indicações), e outras 4 gastrectomias foram realizadas entre 39 pacientes com cirurgias prévias sem ressecção (10%). No total, houve 51 ressecções após quimioterapia. Fatores independentes associados à sobrevida global em 60 meses foram: quimioterapia pré-operatória (57,3% vs. 40,7%, p=0,029), taxa de complicação, linfadenectomia D2 e estadiamento primário. Casos iniciais mostraram um melhor resultado no grupo neoadjuvante sem significância estatística (p=0,447), o que foi observado nos tumores mais avançados (p=0,027). A cirurgia ótima foi alcançada em 68,6% do grupo neoadjuvante e 51,9% do grupo de cirurgia de princípio (p=0,030), com sobrevida global semelhante (56,6% vs. 52,4%, p=0,904).

**Conclusões:**

Cirurgia de princípio ótima, seguida de terapia adjuvante, com dissecção D2, é eficaz e não foi estatisticamente inferior ao tratamento neoadjuvante.

## INTRODUCTION

 Despite the recent increase in cardia tumors, the distal stomach remains an important site of gastric adenocarcinoma^16 ,21,23 ,26^. Due to the high rate of late diagnoses, patients’ nutritional status is commonly affected, sometimes with gastric outlet obstruction, which often requires intervention before systemic treatment^5, 6,14, 17^. Patient morbidity, costs, side effects, and variable response rates also hinder the decision for neoadjuvant treatment. In this context, upfront surgery becomes an alternative. From 2013 to 2024, 85.7% of gastric cancer surgeries in the Brazilian public health system were performed within 60 days of the diagnosis, whereas only 52.5% of patients referred for chemotherapy started the treatment within this period^24^ . The objective of this study is to compare upfront resection with neoadjuvant systemic therapy followed by surgery and to identify factors influencing their outcomes. 

## METHODS

 The study is a retrospective analysis of patients with locally advanced gastric adenocarcinoma followed between 2012 and 2020 at a single center, comparing upfront surgery and preoperative chemotherapy treatment. Patients with clinical diagnoses of early tumor (cT1N0), distant metastasis, and stump cancer were initially excluded. After staging laparoscopy or laparotomy, patients with unexpected carcinomatosis or other metastases were also excluded. 

 The treatment percentage in each group was calculated by dividing the total number of resections by the number of indicated patients, excluding cases with unexpected metastasis. Overall survival (OS) at 60 months was compared between the resections performed in an upfront (UPF) surgery setting and surgery following systemic treatment (neoadjuvant [NEO]), including resection cases with initial unsuccessful laparotomy attempts. The proposed treatment for each patient was chosen in routine tumor board meetings, considering tumor characteristics, patient clinical and social characteristics, such as conditions for regular transportation, family support, patient understanding and commitment to therapy. If possible, patients with more advanced tumors were intended to be referred for NEO treatment, but obstructive symptoms and social aspects frequently directed them to surgery. Lymphadenectomy D1 or D2 was defined by the surgeon based on intraoperative feasibility, where D1 was only performed when radical dissection was considered unsafe. Further postoperative analyses were performed, considering "true D2," the cases with dissection of at least stations 8a, 9, and 11p on the pathology report, whereas "true D1" was considered when it involved only perigastric nodes and station 7. Resections with D2 dissection, no residual tumor (no R2), and no complications were considered optimal surgery. As a retrospective study, these analyses did not influence treatment decisions. 

 Tumor staging was based on the TNM (Tumor, Lymph Node, Metastasis) 8th edition^3^ . For clinical staging of lymph nodes, the criteria used by Fukagawa et al.^10^ were applied. Nodes with a long axis=10 mm or a short axis=8 mm on tomography were considered positive. If the tumor depth was not defined by tomography, the endoscopic criteria of 6 cm size for more advanced lesions, used by Tanaguchi et al.^25^ , were applied. First, the entire cohort from both arms was compared, which was also adopted in prospective randomized studies^8,27^. Second, to stratify the comparison between NEO and UPF by stage without the influence of systemic treatment, the clinical classification was used for NEO and the pathological classification for UPF, named the primary stage. NEO clinical stages cE I–II and UPF pathological stages pE IB–II were considered initial tumors. NEO clinical stages III–IVa and UPF pathological stage III were considered more advanced tumors. 

 Perioperative chemotherapy mainly consisted of protocols based on either Capecitabine or Epirubicin (XELOX or ECF). Most patients in the UPF group received postoperative MacDonald or XELOX protocol. Multivisceral surgery was defined by the resection of at least one of the following organs: colon, liver, pancreas, spleen, or diaphragm. The majority of patients (90%) were followed for at least 60 months, and the minimum period was 45 months. All data were obtained from the Esophagogastric and Bariatric Surgery Division of Santa Casa de São Paulo. 

 Patient characteristics were presented through frequencies and percentages for qualitative variables and by mean, median, standard deviation, minimum, and maximum values for quantitative variables. Association between qualitative variables was evaluated by Pearson’s ꭓ^2^ test or Fisher’s exact test (when 25% or more of the expected values were less than 5). The comparison between two groups for quantitative variables was assessed by Student’s t-test or Mann-Whitney test (if the data did not follow a normal distribution). In the overall survival analysis, the time from surgery to death or last information was calculated; patients alive at the end of follow-up were considered "censored." Survival curves were constructed using the Kaplan-Meier method and compared through the log-rank test. Additionally, Cox regression was used to calculate the hazard ratio (HR) and respective 95% confidence intervals. Finally, both unadjusted and adjusted HR values, according to variables of interest, were presented. Binary logistic regression was utilized to evaluate factors related to optimal surgery. Odds ratios (ORs) and 95% confidence intervals (95%CIs) were calculated for each variable of interest and presented through unadjusted and adjusted OR values. The analyses were performed using the statistical software SPSS for Windows v.25 and Stata v.14 for Windows. The study was approved by the Ethics Committee of the Institution (CAAE number 69680423.6.000). 

## RESULTS

**Figure 1:**
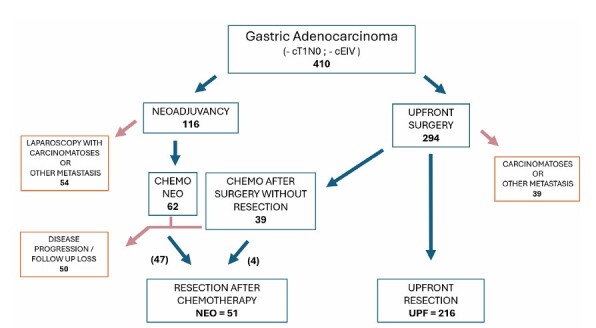
Study flowchart.

 During the study period, 410 patients with locally advanced gastric cancer attended our institution. Two-hundred and ninety-four patients were referred directly to surgery, and 39 of them had unexpected metastasis. Of the remaining 254, upfront resection (UPF) was performed in 216 (85%). In the NEO group with 116 patients, the staging laparoscopy detected metastasis in 54. Out of 62 patients, 47 (76%) underwent gastrectomy after neoadjuvancy, and another four were resected among 39 previous unsuccessful resection attempts (10%). In total, there were 51 resections after chemotherapy (NEO) (Figure 1). 

cT1N0: early gastric câncer; cEIV: distant metastasis, stump cancer, and carcinomatosis; chemo=chemotherapy; NEO: neoadjuvant.

 UPF and NEO had similar mean ages of 61.8 and 58.0 (p=0.05). In both groups, male patients were more frequent. More advanced primary stage (pEIII) corresponded to 72.5% of UPF cases, and in NEO (cEIII and IVa), they were 63.9% of the patients, without statistical significance (p=0.242). The tumor size distribution, based on the number of affected stomach segments, was similar in UPF and NEO. The frequency of subtotal gastrectomies was 79.2% in UPF, with 65.3% of lower-centered tumors, whereas in NEO, subtotal resections represented 47.1% of cases (p<0.001), with 35.3% of the main portion of lesions in the distal stomach. There was no statistically significant difference in Lauren’s histological type, but NEO had 63.9% of diffuse cell adenocarcinoma, more than UPF with 47.1% (p=0.086). Complete pathological response corresponded to 9.8% of NEO patients, and ypN0 was observed in 60.8% of cases. The percentage of D2 lymphadenectomy was 62.0% in UPF and 74.5% in NEO, with no statistically significant difference (p=0.094). Among D2 surgeries, D2 with 8a/9/11p dissection was also similar in UPF and NEO, with 89.6% and 92.1% (p=0.767), as was D1 without 8a/9/11p among D1 surgeries, with 86.6% and 76.9% (p=0.400), respectively. The mean number of resected lymph nodes had no statistically significant difference between the two groups, with 29.1 in UPF and 32.4 in NEO (p=0.066, p>0.05). When analyzed separately by type of lymphadenectomy, numbers were also similar for both D1 (UPF with 20.2 vs. NEO with 20.4 resected nodes, p=0.918, p>0.05), and D2 (UPF with 34.5 vs. NEO with 36.6, p=0.205). Patients, main characteristics are presented in Table 1, and the complete data are presented in Tables 2, 3, and 4. 

**Table 1:** Patients main characteristics.

Characteristics		UPF	NEO	p-value
		n=216	n=51	
Age (years)				
	Mean (SD)	61.8 (12.8)	58.0 (10.9)	0.05
	Median (min–max)	63 (24–92)	57 (30–78)	
Lauren’s type (%)				
	Diffuse	138 (63.9)	24 (47.1)	0.086
	Intestinal	72 (33.3)	25 (49.0)	
	Mixed	6 (2.8)	2 (3.9)	
Primary stage (pUPF x cNEO) (%)				
	Initial	78 (36.1)	14 (27.5)	0.242
	More advanced	138 (63.9)	37 (72.5)	
Lymphadenectomy (%)				
	D1	82 (38.0)	13 (25.5)	0.094
	D2	134 (62.0)	38 (74.5)	
Lymphadenectomy D2—8a/9/11p dissection (%)				
	Absent	14 (10.4)	3 (7.9)	0.767
	Present	120 (89.6)	35 (92.1)	
Lymphadenectomy D1—8a/9/11p dissection (%)				
	Absent	71 (86.6)	10 (76.9)	0.4
	Present	11 (13.4)	3 (23.1)	
D1 resected lymph nodes				
	Mean (SD)	20.3 (10.2)	20.2 (9.2)	0.918
	Median (IQR)	20 (12–27)	18 (14–22)	
D2 resected lymph nodes				
	Mean (SD)	34.5 (14.9)	36,5 (11.9)	0.205
	Median (IQR)	31 (24–43)	34 (29–48)	
(y)pN (%)				
	(y)pN0	45 (20.8)	31 (60.8)	<0.001
	(y)pN+	171 (79.2)	20 (39.2)	
Surgery (%)				
	Subtotal gastrectomy	171 (79.2)	24 (47.1)	<0.001
	Total gastrectomy	45 (20.8)	27 (52.9)	
Multivisceral surgery (%)				
	No	193 (89.4)	46 (90.2)	0.86
	Yes	23 (10.6)	5 (9.8)	
Residual tumor (R2) (%)				
	No	201 (93.1)	50 (98.0)	0.322
	Yes	15 (6.9)	1 (2.0)	
Complications (%)				
	No	179 (82.9)	47 (92.2)	0.098
	Yes	37 (17.1)	4 (7.8)	
Complications – Leaks (%)				
	No	199 (92.1)	51 (100)	0.05
	Yes	17 (7.9)	0	
Clavien-Dindo (%)				
	No complication	179 (82.9)	47 (92.2)	0.265
	I/II	15 (6.9)	2 (3.9)	
	III/IV/V	22 (10.2)	2 (3.9)	
Optimal surgery (%)				
	No	104 (48.1)	16 (31.4)	0.03
	Yes	112 (51.9)	35 (68.6)	

UPF: upfront; NEO: neoadjuvant; SD: standard deviation; min: minimum value; max: maximum value; IQR: interquartile range (Q1–Q3); UPF: upfront; NEO: neoadjuvant; yPN0: negative lymph nodes; ypN1: positive lymph nodes.

**Table 2:** Patient characteristics^2^.

General characteristics			UPF	NEO	p-value
			n=216	n=51	
Age (years)					
	Mean (SD)		61.8 (12.8)	58.0 (10.9)	0.050^*^
	Median (min–max)		63 (24–92)	57 (30–78)	
Sex (%)					
	Female		88 (40.7)	17 (33.3)	0.330^†^
	Male		128 (59.3)	34 (66.7)	
Tumor center gastric segment (%)					
	Low centered		141 (65.3)	18 (35.3)	<0.001^†^
	Middle centered		45 (20.8)	12 (23.5)	
	Upper centered		30 (13.9)	21 (41.2)	
Number of affected segments (%)					
	1		132 (61.1)	37 (72.5)	0.291^†^
	2		68 (31.5)	12 (23.5)	
	3		16 (7.4)	2 (3.9)
Lauren’s type (%)					
	Diffuse		138 (63.9)	24 (47.1)	0.086^†^
	Intestinal		72 (33.3)	25 (49.0)	
	Mixed		6 (2.8)	2 (3.9)	
Primary stage (pUPF x cNEO) (%)					
	Initial		78 (36.1)	14 (27.5)	0.242^†^
	More advanced		138 (63.9)	37 (72.5)	
Pathological stage					
	Complete response		0	5	0.001^†^
		IA	0	9	
		IB	17	9	
		IIA	25	5	
		IIB	36 (36.1%)	4 (62.7%)	
Initial					
	IIIA		62	11	
	IIIB		54	6	
	IIIC		22 (63,9%)	2 (37,3%)	

UPF: upfront; NEO: neoadjuvant; SD: standard deviation; min: minimum value; max: maximum value.

* Student t-test

† Pearson ꭓ^2^ test

**Table 3:** Patient characteristics of lymphadenectomy and lymph nodes.

Lymphadenectomy characteristics		UPF	NEO	p-value
Resected lymph nodes				
	Mean (SD)	29.1 (15.0)	32.4 (13.3)	0.066^*^
	Median (min–max)	27 (2–86)	32 (8–56)	
Affected lymph nodes				
	Mean (SD)	5.9 (7.5)	2.8 (5.5)	<0.001^*^
	Median (min–max)	4 (0–51)	0 (0–25)	
Lymphadenectomy (%)				
	D1	82 (38.0)	13 (25.5)	0.094^†^
	D2	134 (62.0)	38 (74.5)	
Lymphadenectomy D2 – 8a/9/11p dissection (%)				
	Absent	14 (10.4)	3 (7.9)	0.767^‡^
	Present	120 (89.6)	35 (92.1)	
Lymphadenectomy D1 – 8a/9/11p dissection (%)				
	Absent	71 (86.6)	10 (76,9)	0.400^‡^
	Present	11 (13.4)	3 (23.1)	
D1 resected lymph nodes				
	Mean (SD)	20.3 (10.2)	20.2 (9.2)	0.918^*^
	Median (IQR)	20 (12–27)	18 (14–22)	
D2 resected lymph nodes				
	Mean (SD)	34.5 (14.9)	36.5 (11.9)	0.205^*^
	Median (IQR)	31 (24–43)	34 (29–48)	
(y)pN (%)				
	(y)pN0	45 (20.8)	31 (60.8)	<0.001^†^
	(y)pN+	171 (79.2)	20 (39.2)	

UPF: upfront; NEO: neoadjuvant; SD: standard deviation; min: minimum value; max: maximum value; IQR: interquartile range (Q1–Q3); yPN0: negative lymph nodes; ypN1: positive lymph nodes.

* Mann-Whitney test

† Pearson ꭓ^2^ test

‡ Fisher’s exact test

**Table 4:** Patients surgical characteristics.

Surgical characteristics			UPF	NEO	p-value
			n=216	n=51	
Surgery (%)					
	Subtotal gastrectomy		171 (79.2)	24 (47.1)	<0.001^*^
	Total gastrectomy		45 (20.8)	27 (52.9)	
Multivisceral surgery (%)					
	No		193 (89.4)	46 (90.2)	0.860^*^
	Yes		23 (10.6)	5 (9.8)	
		Pancreas	0	3 (5.9)	0.007^†^
		Liver	2 (0.9)	2 (3.9)	0.166^†^
		Colon	7 (32)	3 (5.9)	0.409^†^
		Spleen	11 (5.1)	2 (3.9)	0.999^†^
		Diaphragm	3 (1.4)	0	0.999^†^
Residual tumor (R2) (%)					
	No		201 (93.1)	50 (98.0)	0.322^†^
	Yes		15 (6.9)	1 (2.0)	
Bleeding (ml)					
	Mean (SD)		263.9 (149.3)	371.3 (263.2)	0.001^‡^
	Median (min-max)		220 (50–780)	350 (50–1500)	
Surgery duration (min)					
	Mean (SD)		209.9 (67.0)	238.0 (65.3)	0.016^‡^
	Median (min-max)		210 (45–390)	240 (140–480)	
Complications (%)					
	No		179 (82.9)	47 (92.2)	0.098^*^
	Yes		37 (17.1)	4 (7.8)	
Complications – Leaks (%)					
	No		199 (92.1)	51 (100)	0.050^†^
	Yes		17 (7.9)	0	
Clavien-Dindo (%)					
	No complication		179 (82.9)	47 (92.2)	0.265^†^
	I/II		15 (6.9)	2 (3.9)	
	III/IV/V		22 (10.2)	2 (3.9)	

UPF: upfront; NEO: neoadjuvant; SD: standard deviation; min: minimum value; max: maximum value.

* Pearson ꭓ^2^ test

† Fisher’s exact test

‡ Mann-Whitney test

 Considering all resected patients, the independent prognostic factors for overall survival at 60 months (OS) were: preoperative treatment, where NEO had better outcomes, with 57.3% compared to 40.7% in the UPF group (HR=0.60, p=0.029, p<0.05); complication rate (HR=1.99, p=0.001, p<0.05); D2 lymphadenectomy (HR=0.63, p=0.006, p<0.05); and primary stage (HR=1.62, p=0.010, p<0.05). Table 5 and 6 show the main variables associated with OS. UPF and NEO groups demonstrated no statistically significant difference in the survival curves for initial tumors (p=0.447, p>0.05), which was observed in the more advanced tumors group (p=0.027, p<0.05). 

**Table 5:** Patients’ complications and postoperative staging.

Variable		HR non-adjusted (95%CI)	p-value	HR adjusted (95%CI)	p-value
Conduct					
	Upfront	1		1	
	Neoadjuvancy	0.57 (0.36–0.90)	0.017	0.60 (0.38–0.95)	0.03
Primary stage					
	Initial	1		1	
	More advanced	1.65 (1.14–2.37)	0.007	1.62 (1.12–2.34)	0.01
Lymphadenectomy					
	D1	1		1	
	D2	0.60 (0.43–0.83)	0.002	0.63 (0.45–0.87)	0.005
Complications					
	No	1		1	
	Yes	2.19 (1.47–3.25)	<0.001	2.04 (1.37–3.03)	<0.001
Pathological stage					
	0–II	1			
	III	2.25 (1.58–3.20)	<0.001		
Angiolymphatic invasion					
	No	1			
	Yes	2.12 (1.37–3.28)	0.001		
Perineural invasion					
	No	1			
	Yes	1.79 (1.23–2.61)	0.002		
Age (years)		1.02 (1.01–1.04)	<0.001		
Variable		HR non adjusted (95%CI)		p-value	
Gastrectomy					
	Subtotal	1			
	Total	1.39 (0.99–1.97)		0.059	
Lauren’s type					
	Diffuse	1			
	Intestinal	0.79 (0.56–1.11)		0.171	
	Mixed	0.97 (0.36–2.62)		0.944	
Multivisceral surgery					
	No	1			
	Yes	1.29 (0.78–2.13)		0.328	
Tumor center					
	Lower	1			
	Middle	1.06 (0.72–1.58)		0.76	
	Upper	0.93 (0.60–1.43)		0.733	
Bleeding (mL)		1.001 (1.000–1.002)		0.207	
Surgery duration (min)		0.998 (0.996–1.001)		0.149	
Sex					
	Female	1			
	Male	0.94 (0.68–1.31)		0.728	

HR: hazard ratio; 95%CI: 95% confidence interval; yPN0: negative lymph nodes; ypN1: positive lymph nodes.

**Table 6:** Patients characteristics of upfront surgery and neoadjuvancy.

Variable		Mean survival time (months)	Overall survival probability			p-value
			1 year	3 years	5 years	
Upfront (n=216)						
Lymphadenectomy (%)						
	D1	27	63.10	30.90	28.30	<0.001
	D2	39.5	80.60	58.00	48.30	
Gastrectomy (%)						
	Subtotal	36.5	78.30	51.00	43.30	0.043
	Total	28.4	57.80	35.60	30.80	
Complications (%)						
	No	37.7	78.70	52.60	43.90	<0.001
	Yes	20.8	51.40	24.30	24.30	
Complications (%)						
	No	37.7	78.70	52.60	43.90	<0.001
	Yes	20.8	51.40	24.30	24.30	
pN (%)						
	pN0	43.4	82.20	71.10	59.50	0.008
	pN+	32.5	71.80	41.50	35.60	
Primary stage (pathological) (%)						
	IB-II	41	79.50	64.10	54.20	0.005
	III	31.2	70.80	38.40	33.00	
Optimal surgery (%)						
	No	26.1	60.40	30.20	28.10	<0.001
	Yes	42.9	86.60	64.00	52.40	
Neoadjuvancy (n=51) (%)						
Lymphadenectomy						
	D1	47.8	92.30	76.90	68.40	0,369
	D2	42.3	81.60	65.70	57.50	
Gastrectomy (%)						
	Subtotal	48.2	91.70	79.20	65.20	0,239
	Total	39.7	77.80	59.30	47.60	
Complications (%)						
	No	43.6	85.10	68.00	59.00	0,81
	Yes	45	75.00	75.00	37.50	
ypN (%)						
	ypN0	52.5	90.30	87.10	83.70	<0,001
	ypN+	29.9	75.00	39.40	15.00	
Primary stage (clinical) (%)						
	I*/II	47.6	92.90	71.40	64.30	0,532
	III/IVa	42.3	81.10	67.60	54.30	
Optimal surgery (%)						
	No	46.4	87.50	75.00	60.60	0,646
	Yes	42.5	82.90	65.60	56.60	
Pathological stage (%)						
	0–II	52.5	90.60	90.60	73.20	<0,001
	III	29	73.70	30.70	30.70	

yPN0: negative lymph nodes ; ypN1: positive lymph nodes ; pN: postoperative lymph nodes.

 The UPF group had a lower bleeding rate and shorter procedures, but it demonstrated higher complication rates of 17.1% vs. 7.8% without a statistically significant difference (p=0.098, p>0.05). Among all UPF patients, the complications were characterized by 7.9% of anastomotic or stapler leaks and a 10.2% rate of Clavien-Dindo III or higher, including a 6% mortality rate, which was not seen in NEO (0%). Both UPF and NEO had around 10% of multivisceral resections, but with different distributions of affected organs. UPF had more R2 surgeries, with 6.9% vs. 2.0%, without statistical significance (p=0.322, p>0.05). 

 Lymphadenectomy showed different outcomes in UPF, as in this group, D1 dissection had an OS of 28.3 vs. 48.3% with D2 dissection (p<0.001). The difference was not as large in the NEO group, with OS of 68.4% for D1 and 57.5% for D2 surgeries (p=0.369, p>0.05). Consequently, UPF and NEO had similar survival curves with D2 dissection (p=0.586, p>0.05) and different curves with D1 dissection (p=0.011, p<0.05). Optimal surgery represented 68.6% of NEO and 51.9% of UPF (p=0.030, p>0.05) and had similar OS curves in both groups (p=0.904, p>0.05). 

 We analyzed a UPF group of 210 patients, examining preoperatively predictable variables in relation to optimal surgery. Independent associated variables were age (less than 62 years), total gastrectomy, no multivisceral surgery, no three-segment tumor, and diffuse type (Table 7). Additionally, we assessed variables linked to increased complications in patients who underwent radical surgery (D2 lymphadenectomy without R2 resection, n=130) (Table 8). 

**Table 7:** Preoperative predictable variables related to upfront optimal surgery.

Variable		OR non-adjusted (95%CI)	p-value	OR adjusted (95%CI)	p-value
Age (ref.>62 years)^*^		1		1	
	≤62 years	3.53 (2.00–6.24)	<0.001	3.73 (2.00–6.97)	<0.001
Sex (ref. Male)		1			
Female		1.31 (0.76–2.28)	0.337		
Stage (ref. More advanced)		1		1	
	Initial	1.47 (0.84–2.59)	0.182	1.66 (0.88–3.12)	0.118
Gastrectomy (ref. Subtotal)		1		1	
	Total	1.79 (0.90–3.56)	0.095	3.40 (1.28–9.08)	0.014
Multivisceral (ref. Yes)		1		1	
	No	1.68 (0.68–4.11)	0.259	3.38 (1.06–10.73)	0.039
Main segment (ref. L)		1			
	Segment M	1.42 (0.72–2.83)	0.314		
	Segment U	1.06 (0.47–2.35)	0.894		
Number of segments (ref. 1)		1		1	
	2	1.65 (0.91–3.02)	0.102	1.27 (0.64–2.49)	0.497
	3	0.82 (0.29–2.32)	0.703	0.20 (0.05–0.84)	0.028
Histology (ref. Intestinal)		1		1	
	Diffuse	1.93 (1.08–3.44)	0.026	2.04 (1.07–3.91)	0.031
Angiolymphatic inv. (ref. Yes)		1			
	No	1.22 (0.61–2.46)	0.579		
Perineural inv. (ref. Yes)		1			
	No	0.75 (0.40–1.39)	0.355		

N: 210 (six cases with mixed Lauren’s type were excluded); OR: odds ratio; 95%CI: 95% confidence interval.

* Median age=62.

**Table 8:** Preoperative predictable variables related to absence of complications in radical upfront surgery (D2 lymphadenectomy without R2 resection).

Variable		OR non-adjusted (95%CI)	p-value	OR adjusted (95%CI)	p-value
Age (ref.>62 years)^*^		1		1	
	≤62 years	7.28 (2.28–23.30)	0.001	9.57 (2.76–33.20)	<0.001
Sex (ref. Male)		1			
Female		3.21 (1.01–10.23)	0.048		
Stage (ref. More advanced)		1			
	Initial	1.62 (0.58–4.52)	0.361		
Gastrectomy (ref. Subtotal)		1			
	Total	1.37 (0.42–4.43)	0.604		
Multivisceral (ref. Yes)		1			
	No	3.74 (1.10–12.68)	0.034		
Main segment (ref. L)		1		1	
	Segment M	0.34 (0.12-0.96)	0.042	0.22 (0.07–0.71)	0.012
	Segment U	0.58 (0.14-2.45)	0.458	0.62 (0.13–2.82)	0.533
Number of segments (ref. 1)		1			
	2	1.59 (0.52–4.84)	0.102		
	3	0.45 (0.10–2.00)	0.703		
Histology (ref. Intestinal)		1			
	Diffuse	1.78 (0.66–4.76)	0.254		
Angiolymphatic inv. (ref. Yes)		1			
	No	4.81 (0.61–37.87)	0.136		
Perineural inv. (ref. Yes)		1			
	No	0.93 (0.31–2.80)	0.895		

N: 130 (three cases with mixed Lauren’s type were excluded); OR: odds ratio; 95%CI: 95% confidence interval.

* Median age=62.

## DISCUSSION

 Since the publication of the MAGIC trial in 2006^8^ , preoperative chemotherapy for gastric adenocarcinoma has improved, and its benefit in Western countries was further confirmed by FLOT trial in 2019^1,2^. Other Brazilian studies were compatible with our analysis, and they have also shown the advantage of preoperative chemotherapy in those patients that complete treatment, despite problems with patients’ poor conditions and health care systems^7,11,18^. Gastric outlet obstruction is one of the most important factors leading to the decision for upfront surgery in gastric cancer^5,6 ,14,17^, and it has been described as a worse prognostic factor for non-metastatic cases^6,14,17^. Unsurprisingly, our study identified more distal tumors, reflecting the necessity of intervention in patients with gastric outlet obstruction. The response to NEO therapy, considering the ypN0 rate and the absence of residual tumor percentage, was comparable to the literature, ranging from 31^8^ to 59%^1^ and from 3.0^26^ to 16%^1^ respectively. As expected, this response to preoperative chemotherapy was more beneficial for more advanced tumors, which contributes to validate the adopted stratification criteria. 

 In our study, UPF presented a higher complication rate than NEO, including Clavien-Dindo III or higher, which was not seen in other studies^8,11,20,27^. Although not all preoperative data were available, UPF patients might have had a worse clinical condition and nutritional status, due to obstructive disease. This may also be caused by the selection effect of neoadjuvancy. Patients operated on in the NEO group had already managed to overcome chemotherapy toxicity to be fit for surgery, representing a less vulnerable population, which is suggested by the lower percentage of patients reaching surgery in this group. This difference was also present in prospective trials^8,27^. 

 D2 dissection was an important prognostic factor in the UPF group, which was not seen in the NEO. The equivalent quality of lymphadenectomy was verified by the similar proportion of nodes resected during D1 and D2 surgeries in both groups, and they also had a similar percentage of D2 with 8a/9/11p and D1 without 8a/9/11p, which was adopted to verify more precisely "true" D1 and D2 lymphadenectomy postoperatively. Previous studies have contributed to this discussion in the upfront surgery setting^9 ,12,22^, but there are few studies concerning surgery radicality after the rise of preoperative chemotherapy. 

 The parameters used for optimal surgery are contemplated in the study of benchmarks for adenocarcinoma gastrectomies^19^ . When patients underwent optimal surgery, there were no statistical survival differences between UPF and NEO, which contributes to the discussion about the selection of patients who benefit from preoperative chemotherapy. In addition, the lower percentage of patients reaching surgery in NEO is also relevant, pointing out the importance of the upfront alternative. It may be considered in a high incidence of obstructive distal tumors scenario, but also in the context of primary initial tumors, where UPF was also demonstrated to be not statistically inferior to NEO. This reflects the need for more prospective studies with primary stage stratification. Nevertheless, upfront surgeries had more morbidity, and together with the necessity of D2 dissection, they might become riskier. 

 The analysis of preoperatively predictable variables within the UPF group reveals which patients had a better chance of optimal upfront surgery. While tumor location and stage were not statistically significant, three-segment tumors showed a worse outcome. Total gastrectomy, the absence of multivisceral surgery, and age were also independent factors for optimal surgery. It is important to consider that performing radical surgery (D2 lymphadenectomy without R2 resection) involves the surgeon’s intraoperative decisions. Objectively, we also analyzed the variables related to complication rate exclusively in patients who underwent radical surgery. The only independent factor that remained statistically significant in both analyses was age younger than 62 years. This suggests a greater relevance of the patient’s clinical condition over the tumor characteristics themselves for the likelihood of optimal procedures when focusing solely on the upfront surgery setting. However, this must be interpreted carefully, as larger lesions may indirectly affect a patient’s overall status, and older patients may also have poorer conditions for receiving chemotherapy, necessitating a tailored decision-making process. In the literature, more advanced stages and total gastrectomies are associated with a higher incidence of complications^4,15^, while middle segment tumors are linked with better prognoses, though the association with complications is not widely described^13^ . These differences from our results warrant further investigation with a larger patient cohort and more comprehensive preoperative factors, but this certainly provides a promising direction for future research. 

 Regarding the implications for practice, we acknowledge that the current standard in many Western countries favors NEO treatment for advanced tumors when feasible. However, the reality of needing to perform upfront surgery, whether due to obstructive symptoms or patients’ poor clinical and support conditions for receiving chemotherapy, necessitates a deeper understanding of this approach. Our study aimed to explore these scenarios, and our conclusions about optimal surgery point out that by verifying its likelihood preoperatively, it might be possible to better select patients for systemic treatment to avoid the risk of progression and side effects. Conversely, another practical conclusion is the need to identify unexpected intraoperative findings that hinder safe, optimal surgery. In this situation, surgeons may consider not advancing to resections, evaluating nutritional procedures, such as gastroenterostomies or jejunostomies, and directing the patient to NEO treatment. 

 As a retrospective study, our analyses had some limitations. Imprecise clinical staging, which is a major concern in gastric cancer treatment decisions, is also expected in our study, and some staging exams were performed at different institutions before the patients were referred to our service. The preoperative chemotherapy started mainly in 2017 in our institution, leading to a smaller group of these patients. During the study period, systemic protocols were changed, with variable complete treatment rates, which corresponds to a bias. Comorbidities and body mass index information were frequently missing and could not be used. 

 We debated the use of propensity score matching, and we concluded that it might not be the most suitable approach given the smaller number of patients in the NEO arm, which could limit statistical power. Key baseline characteristics (age, primary stage, histological type, lymphadenectomy, multivisceral surgery, and residual tumor rate) were not statistically different between the groups. The higher incidence of distal tumors and subtotal gastrectomies in the UPF group, within a context of more advanced stages, might suggest a higher proportion of obstructive tumors and patients with poorer base line status. However, even in this context, the outcomes of optimal upfront surgery were similar to those of NEO treatment. 

## CONCLUSIONS

 Optimal upfront surgery followed by adjuvant therapy, particularly with D2 dissection, is effective and was not statistically inferior to NEO treatment. 

## Data Availability

The datasets generated and/or analyzed during the current study are available from the corresponding author upon reasonable request.

## References

[1] Al-Batran SE, Hofheinz RD, Pauligk C, Kopp HG, Haag GM, Luley KB (2016). Histopathological regression after neoadjuvant docetaxel, oxaliplatin, fluorouracil, and leucovorin versus epirubicin, cisplatin, and fluorouracil or capecitabine in patients with resectable gastric or gastro-oesophageal junction adenocarcinoma (FLOT4-AIO): results from the phase 2 part of a multicentre, open-label, randomised phase 2/3 trial. Lancet Oncol.

[2] Al-Batran SE, Homann N, Pauligk C, Goetze TO, Meiler J, Kasper S (2019). Perioperative chemotherapy with fluorouracil plus leucovorin, oxaliplatin, and docetaxel versus fluorouracil or capecitabine plus cisplatin and epirubicin for locally advanced, resectable gastric or gastro-oesophageal junction adenocarcinoma (FLOT4): a randomised, phase 2/3 trial. Lancet.

[3] Amin MB, Edge SB, Gress DM, Meyer LR (2017). American Joint Committee on Cancer.

[4] Baiocchi GL, Giacopuzzi S, Reim D, Piessen G, Costa PMD, Reynolds JV (2020). Incidence and grading of complications after gastrectomy for cancer using the GASTRODATA registry: a European retrospective observational study. Ann Surg.

[5] Blumenthaler AN, Ikoma N, Blum M, Das P, Minsky BD, Mansfield PF (2020). Relationship between initial management strategy and survival in patients with gastric outlet obstruction due to gastric cancer. J Surg Oncol.

[6] Chen JH, Wu CW, Lo SS, Li AFY, Hsieh MC, Shen KH (2007). Outcome of distal gastric cancer with pyloric stenosis after curative resection. Eur J Surg Oncol.

[7] Coimbra FJF, Jesus VHF, Ribeiro HSC, Diniz AL, Godoy AL, Farias IC (2019). Impact of ypT, ypN, and adjuvant therapy on survival in gastric cancer patients treated with perioperative chemotherapy and radical surgery. Ann Surg Oncol.

[8] Cunningham D, Allum WH, Stenning SP, Thompson JN, Van de Velde CJ, Nicolson M (2006). Perioperative chemotherapy versus surgery alone for resectable gastroesophageal cancer. N Engl J Med.

[9] Degiuli M, Reddavid R, Tomatis M, Ponti A, Morino M, Sasako M (2021). D2 dissection improves diseasespecific survival in advanced gastric cancer patients: 15year follow-up results of the Italian Gastric Cancer Study Group D1 versus D2 randomised controlled trial. Eur J Cancer.

[10] Fukagawa T, Katai H, Mizusawa J, Nakamura K, Sano T, Terashima M (2018). A prospective multi-institutional validity study to evaluate the accuracy of clinical diagnosis of pathological stage III gastric cancer (JCOG1302A). Gastric Cancer.

[11] Hong S, Pereira MA, Victor CR, Gregório JVA, Zilberstein B, Ribeiro U (2023). Preoperative chemotherapy versus upfront surgery for advanced gastric cancer: a propensity score matching analysis. Arq Bras Cir Dig.

[12] Maruyama K, Kaminishi M, Hayashi K, Isobe Y, Honda I, Japanese Gastric Cancer Association Registration Committee (2006). Gastric cancer treated in 1991 in Japan: data analysis of nationwide registry. Gastric Cancer.

[13] Ji X, Yan Y, Bu ZD, Li ZY, Wu AW, Zhang LH (2017). The optimal extent of gastrectomy for middle-third gastric cancer: distal subtotal gastrectomy is superior to total gastrectomy in short-term effect without sacrificing long-term survival. BMC Cancer.

[14] Jiao X, Wang Y, Qu X, Qu J, Wang X (2021). Effects of preoperative pyloric stenosis on outcomes and nutritional status in 73 patients following curative gastrectomy for gastric cancer: a retrospective study from a single center. Med Sci Monit.

[15] Lian B, Chen J, Li Z, Ji G, Wang S, Zhao Q (2020). Risk factors and Clavien-Dindo classification of postoperative complications after laparoscopic and open gastrectomies for gastric cancer: a single-center, large sample, retrospective cohort study. Cancer Manag Res.

[16] Martel C, Forman D, Plummer M (2013). Gastric cancer: epidemiology and risk factors. Gastroenterol Clin North Am.

[17] Park SH, Mok YJ, Kim JH, Park SS, Kim SJ, Kim CS (2009). Clinical significance of gastric outlet obstruction on the oncologic and surgical outcomes of radical surgery for carcinoma of the distal stomach. J Surg Oncol.

[18] Ramos MFKP, Pereira MA, Albuquerque AF, Viana EF, Costa WL, Sanches SRA (2025). Implementation of the recommendations of the II Brazilian Consensus On Gastric Cancer in clinical practice: a multicenter study of the Brazilian Gastric Cancer Association. Arq Bras Cir Dig.

[19] Schneider MA, Kim J, Berlth F, Sugita Y, Grimminger PP, Sano T (2024). Defining benchmarks for total and distal gastrectomy: global multicentre analysis. Br J Surg.

[20] Schuhmacher C, Gretschel S, Lordick F, Reichardt P, Hohenberger W, Eisenberger CF (2010). Neoadjuvant chemotherapy compared with surgery alone for locally advanced cancer of the stomach and cardia: European Organisation for Research and Treatment of Cancer randomized trial 40954. J Clin Oncol.

[21] Seferian G, Zamot S, Castro OAP, Zanon CC, Bafutto AAF, Pochini CC (2021). Comparison of clinical behavior of cardia and antral adenocarcinomas: revisiting an old issue in Brazil. J Surg.

[22] Songun I, Putter H, Kranenbarg EMK, Sasako M, van de Velde CJH (2010). Surgical treatment of gastric cancer: 15-year follow-up results of the randomised nationwide Dutch D1D2 trial. Lancet Oncol.

[23] Sung H, Ferlay J, Siegel RL, Laversanne M, Soerjomataram I, Jemal A (2021). Global Cancer Statistics 2020: GLOBOCAN estimates of incidence and mortality worldwide for 36 cancers in 185 countries. CA Cancer J Clin.

[24] Brasil, Ministério da Saúde Datasus.

[25] Taniguchi K, Ota M, Yamada T, Serizawa A, Noguchi T, Amano K (2019). Staging of gastric cancer with the Clinical Stage Prediction score. World J Surg Oncol.

[26] Thrift AP, Wenker TN, El-Serag HB (2023). Global burden of gastric cancer: epidemiological trends, risk factors, screening and prevention. Nat Rev Clin Oncol.

[27] Ychou M, Boige V, Pignon JP, Conroy T, Bouché O, Lebreton G (2011). Perioperative chemotherapy compared with surgery alone for resectable gastroesophageal adenocarcinoma: an FNCLCC and FFCD multicenter phase III trial. J Clin Oncol.

