# ERRATUM: Optimal upfront surgery for gastric adenocarcinoma. Real life situation in Brazil. Results comparable to neoadjuvant treatment

**DOI:** 10.1590/0102-67202025000057e1924-ER

**Published:** 2026-05-25

In the manuscript "Optimal upfront surgery for gastric adenocarcinoma. Real life situation in Brazil. Results comparable to neoadjuvant treatment", DOI: https://doi.org/10.1590/0102-67202025000055e1924, published in the ABCD Arq Bras Cir Dig 2025;38:e1924.

Page 3, second column, item "Results", paragraph 2.

**Where it reads:**

More advanced primary stage (pEIII) corresponded to 72.5% of UPF cases, and in NEO (cEIII and IVa), they were 63.9% of the patients, without statistical significance (p=0.242).

**It should read:**

More advanced primary stage (pEIII) corresponded to 63.9% of UPF cases, and in NEO (cEIII and IVa), they were 72.5% of the patients, without statistical significance (p=0.242, p>0.05).

**Where it reads:**

There was no statistically significant difference in Lauren's histological type, but NEO had 63.9% of diffuse cell adenocarcinoma, more than UPF with 47.1% (p=0.086).

**It should read:**

There was no statistically significant difference in Lauren's histological type, but UPF had 63.9% of diffuse cell adenocarcinoma, more than NEO with 47.1% (p=0.086, p>0.05).

